# Onset Patterns and Course of Myalgic Encephalomyelitis/Chronic Fatigue Syndrome

**DOI:** 10.3389/fped.2019.00012

**Published:** 2019-02-05

**Authors:** Lily Chu, Ian J. Valencia, Donn W. Garvert, Jose G. Montoya

**Affiliations:** Stanford ME/CFS Initiative, Division of Infectious Diseases and Geographic Medicine, Stanford University School of Medicine, Stanford, CA, United States

**Keywords:** chronic fatigue syndrome, myalgic encephalomyelitis, epidemiology, onset, course, systemic exertion intolerance disease, natural history

## Abstract

**Background:** Epidemiologic studies of myalgic encephalomyelitis/ chronic fatigue syndrome (ME/CFS) have examined different aspects of this disease separately but few have explored them together.

**Objective:** Describe ME/CFS onset and course in one United States-based cohort.

**Methods:** One hundred and fifty subjects fitting Fukuda 1994 CFS criteria completed a detailed survey concerning the initial and subsequent stages of their illness. Descriptive statistics, graphs, and tables were used to illustrate prevalence and patterns of characteristics.

**Results:** The most common peri-onset events reported by subjects were infection-related episodes (64%), stressful incidents (39%), and exposure to environmental toxins (20%). For 38% of subjects, more than 6 months elapsed from experiencing any initial symptom to developing the set of symptoms comprising their ME/CFS. Over time, the 12 most common symptoms persisted but declined in prevalence, with fatigue, unrefreshing sleep, exertion-related sickness, and flu-like symptoms declining the most (by 20–25%). Conversely, cognitive symptoms changed the least in prevalence, rising in symptom ranking. Pregnancy, menopause, and menstrual cycles exacerbated many women's symptoms. Fatigue-related function was not associated with duration of illness or age; during the worst periods of their illness, 48% of subjects could not engage in any productive activity. At the time of survey, 47% were unable to work and only 4% felt their condition was improving steadily with the majority (59%) describing a fluctuating course. Ninety-seven percent suffered from at least one other illness: anxiety (48%), depression (43%), fibromyalgia (39%), irritable bowel syndrome (38%), and migraine headaches (37%) were the most diagnosed conditions. Thirteen percent came from families where at least one other first-degree relative was also afflicted, rising to 27% when chronic fatigue of unclear etiology was included.

**Conclusions:** This paper offers a broad epidemiologic overview of one ME/CFS cohort in the United States. While most of our findings are consistent with prior studies, we highlight underexamined aspects of this condition (e.g., the evolution of symptoms) and propose new interpretations of findings. Studying these aspects can offer insight and solutions to the diagnosis, etiology, pathophysiology, and treatment of this condition.

## Introduction

Myalgic encephalomyelitis/ chronic fatigue syndrome is a complex, disabling, chronic illness that is estimated to affect from 0.76 to 3.28% (1) of the population worldwide and up to 2.5 million US residents (2). Although the average age of onset is in the 30 s and women are affected at two to three times the rate of men, CFS can occur at any age, also strikes children, and, contrary to its early nickname, “yuppie flu,” may disproportionately affect certain ethnic minorities as well as lower socio-economic classes (2–4). Severe fatigue accompanied by musculoskeletal pain, headaches, sore throat, tender lymph nodes, concentration/ memory difficulties, unrefreshing sleep, exacerbation of these symptoms with minimal physical, or cognitive exertion (termed post-exertional malaise), and orthostatic intolerance results in patients suffering a substantial reduction in function from their pre-illness state (5, 6). Rates of unemployment can be as high as 81% (7) while ~25% of patients may be homebound or bedridden (8). Function and health-related quality of life scores have been shown to be lower than that of patients affected by multiple sclerosis, rheumatoid arthritis, congestive heart failure, and myocardial infarction (9–11). Despite these serious public health implications, after 3 decades, we still do not know what causes ME/CFS nor do we have established objective diagnostic tests or a single FDA-approved treatment (6). Since the median rate of full recovery is only 5% (12), many patients remain ill for years to decades, costing the US ~$18–$54 billion annually from both direct medical costs as well as lost productivity and taxes (13).

Prior studies have documented various aspects of ME/CFS including onset of illness (14, 15), symptoms (6), function (16, 17), course (18–24), co-morbidities (25–29), and family history (30–36). However, these studies have tended to focus on one or a few clinical characteristics. Alternatively, epidemiologic results from one cohort, like the US Centers for Disease Control and Prevention's Wichita ME/CFS group, are dispersed among several articles (21, 23, 37, 38). Consequently, the clinical picture of ME/CFS has had to be pieced together from studies that may have very different subjects or across multiple articles originating from one group of subjects. The few studies that have attempted to give a broad-based overview of one cohort in one article have been based in Europe, Australia, and Japan but not in the United States (39–43). Juxtaposing different aspects of ME/CFS together in one paper might allow researchers and clinicians to see connections among the aspects more easily. Secondly, commonly referenced concepts have not always been clear. For example, many studies classify subjects as having either an “acute”/ “sudden” or “gradual” onset of illness yet most do not define the time period meant by such terms. Researchers, subjects, and article readers might interpret the same term to mean different lengths of time. Third, some aspects of ME/CFS have not been examined in detail. For example, very few papers have examined how symptoms changes over more than a few years and the effect of female reproductive hormonal events on the disease.

The objective of this study was to examine the different dimensions of ME/CFS together and fill in some of these gaps, by characterizing clinical aspects of ME/CFS in detail in one cohort of subjects based in the United States. This study will also serve as a reference for other papers derived from the same cohort (44, 45) exploring relationships among immunological, genetic, microbiological, and clinical characteristics of ME/CFS. Findings from this study may inform clinical care, help generate hypotheses about etiology, pathophysiology, diagnosis, and treatment, and assist in the design and implementation of future studies.

## Methods

From March 2010 to August 2011, 200 ME/CFS subjects were recruited as part of our GESID (Genetic Expression and Immune System Dynamics) study examining the interactions among pathogen presence and load, human leukocyte antigen (HLA) types, and the immune system in ME/CFS. Some subjects originated from Stanford University's ME/CFS Clinic or the Clinic wait list while others were recruited via local support groups and electronic patient forums. All subjects were screened using a standardized telephone interview and included if they fitted Fukuda 1994 CFS criteria, lived in the San Francisco Bay area, were at least 14 years old, were non-pregnant, and had not been exposed to more than 2 weeks of antimicrobials recently. Subjects were excluded from the study if they were affected by an alternative medical or psychiatric condition that could explain their symptoms, suffered from certain immunological conditions, struggled with substance abuse issues in the last year (not including nicotine/caffeine), received an influenza vaccination within the past 4 weeks, or had limited ability communicating in English.

Fatigue severity and impact on function were assessed using the Multi-dimensional Fatigue Inventory −20 (MFI-20) (46, 47) and the Fatigue Severity Scale (FSS) (48). The MFI-20 gives a total score (ranging from 20 to 100) as well as subscores related to 5 dimensions of fatigue (general fatigue, physical fatigue, reduced motivation, reduced activity, and mental fatigue, each ranging from 4 to 20). The FSS total score ranges from 1 to 7 and is an average of the score of 9 individual items, each also rated on a 1–7 scale. For both questionnaires, a higher number indicates a greater impact of fatigue on daily life.

In 2012, to further characterize our subjects, we used the Research Electronic Data Capture (REDCap)^1^ web application to design an online survey covering demographic traits, illness onset, symptoms, illness course, function, patient medical history, family medical history, social history, and medication use. Content, wording, and format of survey items were based on a review of the scientific literature, the authors' clinical/research experiences, and feedback from several patient volunteers. We included a 54-item symptom survey from the DePaul Symptom Questionnaire^2^ (DSQ), which was designed to elicit the wide range of symptoms known to occur in ME/CFS and has been used in multiple other studies. This project was reviewed and approved by the Stanford University Institutional Review Board.

The aforementioned 200 subjects were re-contacted via e-mail or telephone from January 2013 to July 2013 and asked if they wished to participate in the survey. Written consent was obtained. Those expressing interest were given an individualized secured hyperlink to access the survey; if they could not finish it in one session, they were given a code so they could complete it in as many sessions as they needed. A paper version of the survey was also offered to participants who expressed technical or cognitive difficulties with the online survey. After completion, subjects submitted the survey electronically or mailed the survey back to staff.

Next, survey data were stripped of identifying information per the Health Insurance Portability and Accountability Act (HIPAA) Privacy Rule and exported to create a database. We used Microsoft Excel 2016 to generate histograms, scatterplots, and descriptive statistics (frequencies, percentages, mean, medians, standard deviation). To assess for differences between groups and for relationships among variables, we calculated chi-square statistics for categorical variables and Welch's *t*-test for continuous variables using the online program GraphPad^3^ A two-tailed *p*-value of equal or <0.05 was deemed to be statistically significant.

## Results

A total of 150 subjects participated in the survey, equivalent to a 75% response rate. Responders were more likely to be Caucasian (95 vs. 84%, *p* = 0.04), female (80 vs. 66%, *p* = 0.05) and to be affected by sore throats or post-exertional malaise (respectively, 67 vs. 48%, *p* < 0.01; 98 vs. 92%, *p* = 0.04) than non-responders. No statistically significant differences existed otherwise in terms of age, duration of illness, fatigue severity, self-rated physical/cognitive functioning, prevalence of viral onset, or prevalence of minor Fukuda case definition symptoms (data not shown).

Eighty-four percent of the subjects (*n* = 126) completed the survey online. The number of subjects completing each survey item varied as some might not have remembered what had occurred previously or were unsure of their answers. However, for almost every item except one item concerning the onset date of their illness (see next section), more than 92% of the subjects were able to give an answer and only 5 subjects did not complete extensive, contiguous parts of the survey. For all items, statistics were performed based on the number of subjects answering that specific item.

## Demographics

Eighty-one percent of respondents (*n* = 121) were female while 19% were male. An overwhelming 97% of subjects were Caucasian with the remaining subjects identifying themselves as African-American (1%, *n* = 1), Hispanic-American (1%, *n* = 2), and Asian-American (2%, *n* = 3). The median age (standard deviation) of respondents was 53.7 ± 12.4 years (range, 20–75 years of age). Six subjects (4%) could not remember a time in their lives that they had not been sick and 18 subjects could not give an approximate time their illness began. Thus, duration of illness and age of onset could not be determined for these 24 (15%) subjects. Based on available data, the median age of illness onset was 36.6 ± 12.3 years and median duration of illness was 12.5 ± 10.1 years.

## Illness Onset

Most subjects (90%, *n* = 135) could remember a time before they were sick and 85% (*n* = 123) noted a specific time their illness began. When asked if they believed a specific factor precipitated their illness, 88% (*n* = 132) answered affirmatively or possibly.

Although we offered 14 different factors that could be associated with onset, 61% of subjects selected only one or two factors. We chose to group subjects who responded “yes” or “not sure” (vs. a clear “no”) together as illness onset has not been examined in detail and we wanted to include all possibilities. The top five factors selected were infectious illnesses (64%), stress/ major life events (39%, primarily work- or family-related), exposure to chemical/ environmental toxins (20%), recent international travel (19%), and recent domestic travel (17%) (Table 1).

**Table 1:** Factors reported by subjects to be associated with their ME/CFS onset.

**Factor**	**Number of subjects^**a**^**	**Percentage of subjects^**b**^ (%)**
Infectious illness	84	64
Stress or major life event^c^	51	39
Exposure to chemical/environmental toxin^d^	26	20
Recent international travel	25	19
Recent domestic travel	23	17
Other^e^	22	17
Medical injection	13	10
Pregnancy	11	8
Surgery	10	8
Accident	10	8
Consumption of water from questionable source	10	8
Neurologic event	9	7
Cardiac event	8	6
None of the above	5	4
Raw/undercooked dairy, eggs, meat	2	2

a *Subjects responding “yes” or possibly to factor as a precipitant. Subjects could choose more than one factor*.

b *Out of 132 subjects total who noted a precipitating event(s)*.

c *Primarily family and work-related events*.

d *Subjects primarily mentioned environments which might have exposed them to higher levels of various substances. “Mold” was the most common specific answer given*.

e *8 (36%) were infection-related events with all subjects also replying “yes” to infectious illness; remainder included insect bites and other medical events*.

Of the 40 individuals selecting only one factor, 58% chose an infectious event compared to 22% choosing a life event. An “other” category, selected by 17% (*n* = 22), was also included so subjects could write in responses: 36% of these were still infection-related events with the subject also checking the “infectious illness” category. Four percent of subjects cited none of the factors listed and did not write down a specific factor.

Infectious illnesses were further broken down into the type of infectious illness (Table 2). A little over a third of subjects (35%) reported documentation of a specific acute infection; the most common infection-related symptoms were respiratory-related (39%; sore throat, runny nose, cough, etc.) followed closely by constitutional symptoms (33%; fever, chills, etc.). Except for one case, all subjects first fell ill while in the United States.

**Table 2:** Subject-reported infectious events related to ME/CFS-onset.

**Type of infection**	**Number of subjects identifying infection^**a**^**	**Percentage identifying infection^**b**^ (%)**
Respiratory infection (sore throat, runny nose, cough, etc.)	33	39
Documented acute infection (herpes viruses, parvovirus B19, etc.)	29	35
Non-specific infection (fever, chills, sweats, muscle aches, etc.)	28	33
Other	15	18
Abdominal infection (diarrhea, nausea, vomiting, blood in stool, etc.)	10	12
Bladder infection (pain/burning urinating, urinating frequently, feeling of having to urinate urgently, etc.)	4	5
Prostate infection	0	0

a *Out of 84 total respondents endorsing an infectious illness as a precipitating factor for their ME/CFS*.

b *Subjects were permitted to choose more than one type of infectious event. However, 77% chose only one event*.

Half of the subjects (*N* = 13 out of 26) selecting a toxic or chemical trigger did not cite a specific substance but described workplaces, living situations, or hobbies which might have exposed them to unusual levels of various solvents, animal droppings, metals, dust, asbestos, or volatile organic compounds. Six subjects wrote down “mold” but did elaborate further. Subjects traveled widely and no specific portion of the United States nor of the world stood out. Most did not become ill until after returning to the United States. Activities engaged in while traveling included work, visiting family, seeing tourist sites, and participating in outdoor sports. A few noted they recovered from their travel-related illness only to become sick again later (so it was unclear whether ME/CFS was related or not to their travels) while others indicated travel companions did not become sick.

Table 3 shows that the time from the first intimation of illness to becoming consistently sick varied greatly: while 28% endorsed an onset period of a month or less, 38% noted it took over 6 months. Subjects who reported an infectious precipitant were no more likely to develop ME/CFS within 1 month or within 6 months compared to those who noted no infectious precipitant (respectively, 14 vs. 21% and 51 vs. 43%, 0.05 < *p*-value).

**Table 3:** Elapsed time from any initial symptoms to consistent illness.

**Time to onset**	**N**	**%**
Within 24 h	17	12
1–6 days	5	3
7–30 days	19	13
1–6 months	32	22
7–12 months	16	11
1–2 years	11	7
More than 2 years	30	20
Do not know	17	12
No answer	3	–

The second column of Table 4 shows the 12 most prevalent symptoms, out of the 54 elicited by the DSQ, during the first 6 months of illness. Although fatigue/extreme tiredness, endorsed by 97% of subjects, was the most common symptom, five of the remaining symptoms were associated with physical/ cognitive exertion (range 73–85%) and 3 involved cognitive dysfunction (72–76%). Unrefreshing sleep (92%), flu-like feelings (70%), and muscle pain (76%) also figured prominently.

**Table 4:** Prevalence and ranking of the most common 12 symptoms during the first 6 months of illness, after the first 6 months, and at the time of survey^a^.

**Symptom**	**Prevalence first 6 months (rank)^**b**^**	**Prevalence after first 6 months (rank)^**c**^**	**Prevalence at time of survey (rank)**	**Change from beginning of illness to time of survey^**d**^ (%)**
Fatigue/extreme tiredness	97% (1)	86% (1)	76% (1)	−23
Feeling unrefreshed after you wake up in the morning	92% (2)	81% (2)	69% (2)	−23
Physically drained or sick after mild activity	85% (3)	79% (4)	60% (11)	−25
Minimum exercise makes you physically tired	80% (4)	77% (5)	63% (7)	−17
Next day soreness or fatigue after non-strenuous exercise	76% (5)	73% (9)	64% (6)	−12
Problems remembering things	76% (6)	77% (6)	68% (4)	−8
Pain or aching in your muscles	76% (7)	71% (11)	59% (13)	−17
Mentally tired after the slightest effort	75% (8)	76% (7)	61% (9)	−14
Difficulty paying attention	74% (9)	73% (8)	64% (5)	−10
“Dead” or “heavy” feeling after starting to exercise	73% (10)	68% (16)	53% (18)	−20
Difficulty finding the right word/ expressing self	72% (11)	79% (3)	68% (3)	−4
Flu-like symptoms	70% (12)	67% (20)	46% (25)	−24

a *The median length of illness in our sample was 12.5 ± 10.1 years. It was explained to survey respondents that “after first 6 months” meant anytime between that time and the time of the survey. So if someone had been sick for a decade and they suffered from a symptom from years 2 through 5 of their illness, but not at the beginning of their illness or at the time of the survey, they would mark down their answer affirmatively during this period*.

b Subjects were asked if a symptom was present at the specified moment in time. Fifty-four different symptoms were listed with the most common ranked as “1” and the least common as “54.”

c *The prevalence of these symptoms changed over time such that they would no longer be or rise to being among the 12 most common symptoms. For example, “Physically drained or sick after mild activity” was the 3rd most common symptom during the first 6 months but had fallen to the 11th most common by the time of our survey*.

d *Percentage change is absolute, not relative (e.g., for fatigue, 97–76% = 23%). Over time, “absentminded or forgetfulness” (19, 10, 10), “only can focus on one thing at a time” (22, 17, 8), and “sensitivity to noise” (15, 12, 12) moved up to the top 12 most common symptoms (numbers in parentheses refer to change in rank over the 3 time periods)*.

We also asked about the existence of several symptoms that were not included in the DSQ or the Fukuda 1994 criteria but are part of newer criteria like the Systemic Exertion Intolerance Disease/ National Academy of Medicine definition (6), Canadian Consensus Criteria (CCC) (49), and Myalgic Encephalomyelitis-International Consensus Criteria (ME-ICC) (50). Sixty-two percent of subjects reported fainting or near-fainting episodes, 66% were less able to tolerate alcohol compared to their pre-illness state, and 81% felt sick or uncomfortable waiting in lines. All three symptoms are characteristic of orthostatic intolerance. Compared to before the onset of their CFS, 32 and 52% of subjects, respectively, felt they were more prone to viral infections or took a longer time to recover from infections. Eighty-seven percent experienced problems with temperature regulation, especially when the weather was unusually hot or cold.

## Illness Course

For the overwhelming majority of patients (96%, *n* = 141), their illness did not improve with time although different patterns of illness were seen: 14% of subjects believed their illness was constantly worsening; 7%, relapsing-remitting (all symptoms might disappear for a time only to return); 8%, persisting with little change; 59%, fluctuating (symptoms could change in severity but were always present) and 7%, “other” pattern, although the most common response here was analogous to the “fluctuating” pattern with some symptoms worsening while others improved over time. Thirteen percent of subjects reported that they experienced remissions (i.e., no symptoms) of more than a month during their years of illness. The median duration of remission was 7 months with the range being from 1.5 to 240 months.

The symptomology of the illness generally remained unchanged with 9 of the top 12 symptoms present at the beginning of the illness continuing to stay in the top 12 after the initial 6 months and up to the time of this survey more than a decade into illness (Table 4). However, the prevalence of all 12 symptoms decreased over time and three symptoms (“flu-like feelings,” “'dead' or “heavy' feeling after starting to exercise,” “pain or aching in your muscles”) dropped out of the top 12 to be replaced by “absentminded or forgetfulness,” “only can focus on one thing at a time,” and “sensitivity to noise.” Over time, flu-like symptoms, fatigue, unrefreshing sleep, and exertion-related items decreased the most, by between 12 and 25%. For flu-like issues, 55% believed their disappearance to be spontaneously induced whereas for fatigue, exertion-related items and unrefreshing sleep, 72, 50–73, and 90%, respectively, attributed their decline to specific treatments. Cognitive symptoms present at the beginning of the illness tended to persist, declining by only 4–10%.

**Figure 1:**
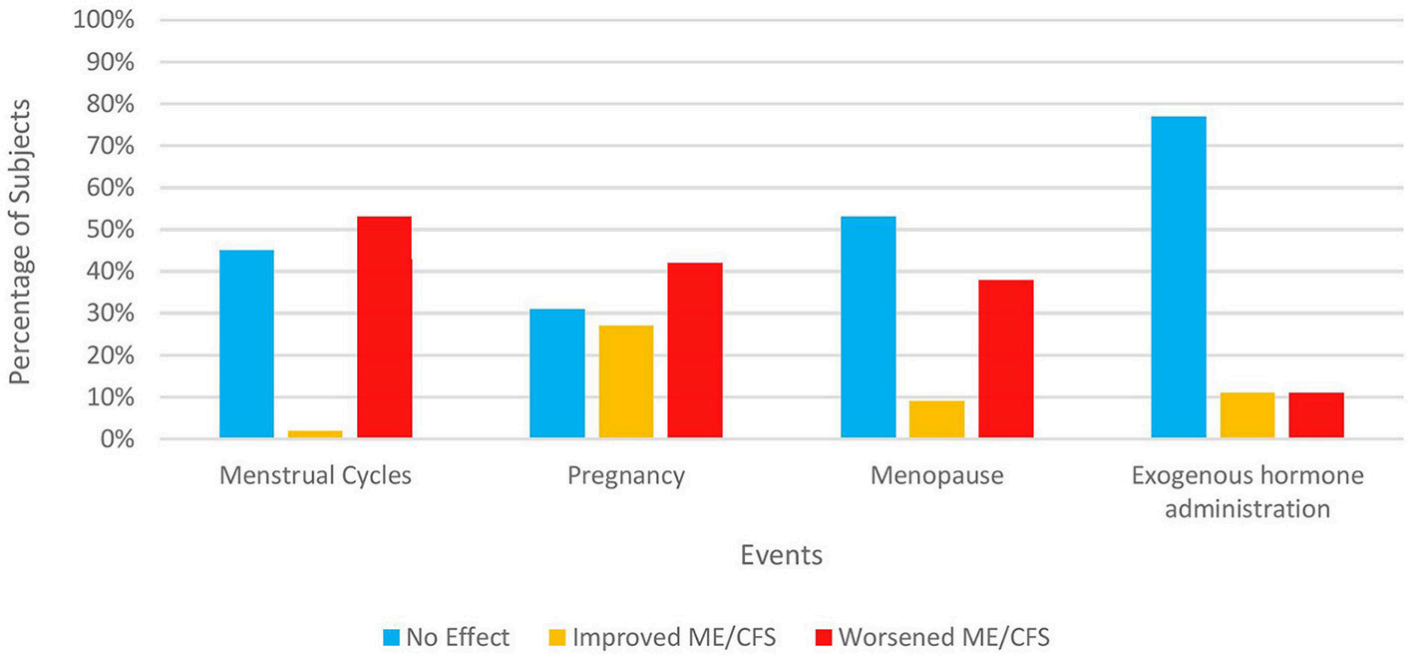
Impact of hormonal events on ME/CFS in women. Only subjects identifying themselves as female were asked these items. 120 out of 121 women responded. The number answering for each event varies depending on both response rate and each woman's circumstances. “Exogenous hormone administration” refers to any form of reproductive hormones (e.g., pills, patch, implants, etc.) taken for contraception, relief of menopausal symptoms, or treatment of any medical condition.

Like the section regarding illness onset, we presented subjects with the same 14 factors and asked which factors they believed might have affected their illness course significantly. The two most-cited factors were infectious illnesses (33%) and stress/ major life events (29%) but a quarter of our subjects cited none of the factors nor wrote in any factors (Table 5). Female subjects were also queried about whether and how specific hormone-related events in their lives affected their symptoms (Figure 1). A significant percentage of women felt that pregnancy (42% overall), menopause (38%), and monthly menstrual cycles (53%) negatively impacted their illness. In contrast, hormone-based contraception and replacement therapy were only cited by 11% as having a deleterious effect with about three-fourths of women citing no effect on their ME/CFS symptoms.

**Table 5:** Subject-reported factors which affected the course of illness.

**Factor**	**Number of subjects**	**Percentage of subjects^**a**^ (%)**
Infectious illness	49	35
Stress or major life event^b^	44	31
None of the above	37	26
Exposure to chemical/environmental toxin^c^	16	11
Other^d^	16	11
Surgery	15	11
Neurologic event	15	11
Cardiac event	15	11
Accident	11	8
Recent domestic travel	8	6
Pregnancy	8	6
Recent international travel	8	5
Medical injection	3	2
Consumption of water from questionable source	1	1
Raw/ undercooked dairy, eggs, meat	1	1

a *Out of 141 respondents*.

b *Work, family, and relationship-related events*.

c *9 out of 16 cited mold; otherwise, a variety of occupational exposures*.

d *8 out of 16 may be infection-related*.

## Function

The 1994 Fukuda CFS criteria requires that “fatigue results in substantial reduction in previous levels of occupational, educational, social, or personal activities.” This level of functional impairment was reflected via the various ways we assessed the impact of ME/CFS on subject's lives. Almost all of our subjects (92%, *n* = 138) believed that the illness had reduced their function by 50% or more; only 15% were able to work more than 30 h a week whereas 47% had been designated as permanently disabled from work.

**Figure 2:**
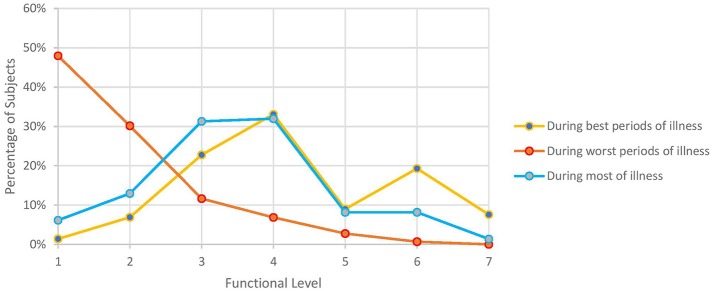
Self-reported functional level during various periods of illness. Numbers 1–7 on x-axis correspond to the following functional levels: (1) I am not able to work, go to school, or do anything, and I am bedridden. (2) I can walk around the house, but I cannot do light housework. (3) I can do light housework, but I cannot work or go to school part-time. (4) I can only work part-time at work or school or on some family responsibilities. (5) I can work or go to school full time, but I have no energy left for anything else. (6) I can work or go to school full time and finish some family responsibilities but I have no energy left for anything else. (7) I can do all work, school, or family responsibilities without any problems with my energy.

Figure 2 illustrates functional level, as assessed by ability to carry out work, school, family, and other responsibilities during various periods of an individual subject's illness. For most of their illness, 82% were unable to work or attend school full-time (functional levels 1–4). During the worst periods, nearly half (48%) were confined to their beds or could not engage in any productive activity (functional level 1). Even during their best periods, only about a third of subjects were able to engage in their work or school full-time, albeit it often still meant they had to sacrifice participating in other aspects of life.

**Figure 3:**
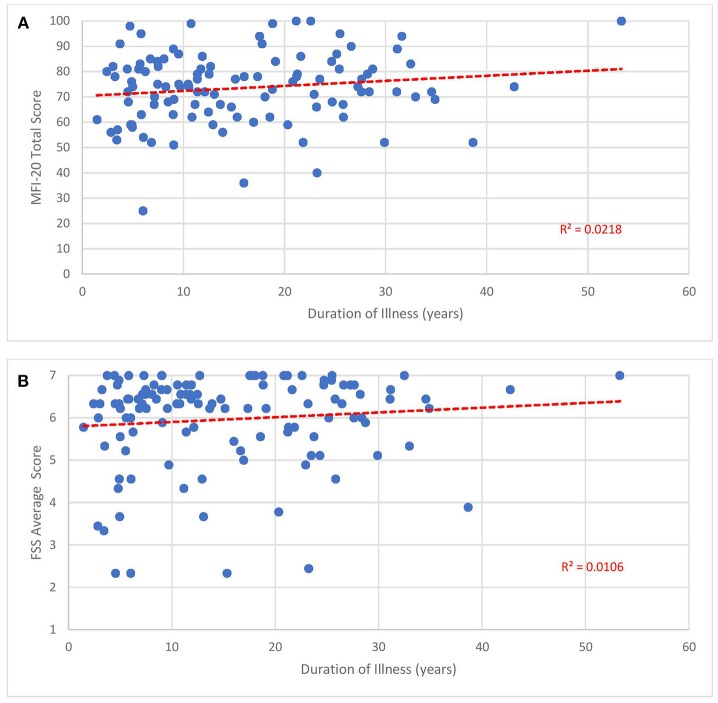
Fatigue questionnaire scores vs. duration of illness. Each point represents one subject. **(A)** Total Multi-dimensional Fatigue Inventory-20 (MFI-20). **(B)** Average Fatigue Severity Scale (FSS) scores.

**Figure 4:**
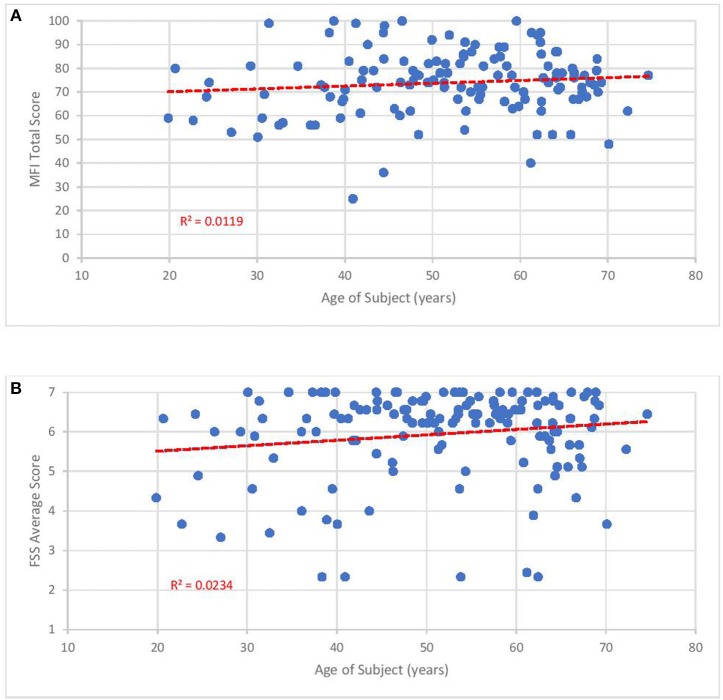
Fatigue questionnaire scores vs. age of subject. Each point represents one subject. **(A)** Total Multi-dimensional Fatigue Inventory-20 (MFI-20). **(B)** Average Fatigue Severity Scale (FSS) scores.

The mean FSS score of our subjects was 5.9 ± 1.1. The mean MFI-20 scores and standard deviations were: total, 73.8 ± 13.6; general, 17.2 ± 3.0; physical, 16.6 ± 3.3; mental, 13.6 ± 4.2; reduced activity, 15.2 ± 4.1; and reduced motivation, 10 ± 4.4. Scatterplots of the total MFI-20 score and total FSS score vs. duration of illness (Figure 3) and age of the subject (Figure 4) show little correlation with R-squared values ranging from 0.0106 to 0.0234.

## Personal Medical History

From a list of 43 different medical and psychiatric conditions, including those purported to frequently co-exist with ME/CFS, subjects indicated which conditions they had been diagnosed with by a healthcare professional. They suffered from a median of 7 ± 4.2 conditions with over 50% of the subjects citing any chronic condition as unresolved. Almost all subjects (97%) suffered from at least one other medical condition and 64% divulged at least one psychiatric condition.

The 15 most prevalent conditions are shown in Table 6: anxiety (48%), depression/ seasonal affective disorder/ dysthymia (43%), fibromyalgia (39%), irritable bowel syndrome (38%), and migraine (37%) comprised the 5 most common chronic diagnoses. Past history of varicella zoster infection and symptomatic infectious mononucleosis episode occurred in 82% and 37% (70% noted Epstein-Barr mononucleosis and 17% cytomegalovirus), respectively, while 27% carried a diagnosis of an autoimmune condition. Cancer afflicted 8% but was not among the top 15 conditions. Only 14% cited any history of post-traumatic stress disorder (data not shown).

**Table 6:** Most common co-morbid medical and psychiatric conditions reported by our subjects with ME/CFS^a^ compared to the general United States population and previously published prevalence among ME/CFS subjects.

**Medical condition**	**Percentage (%) with medical condition ever diagnosed**	**Percentage (%) endorsing condition as active/unresolved**	**Prevalence of medical condition, ME/CFS subjects^**d**^ in other studies (%)**	**Prevalence of medical condition, general US population^**e**^ (%)**
Anxiety	48	67	21–88	18
Depression, seasonal affective disorder, or dysthymia	43	63	17–47	7
Fibromyalgia	39	88	12–91	8
Irritable bowel syndrome	38	76	17–92	10–20
Symptomatic infectious mononucleosis^b^	37	1	39	30–50
Migraine headache	37	63	84	14.2
Any autoimmune condition^c^	27	66	13–27	4.5
Chronic sinusitis	33	71	66	8
Hay fever	30	86	33; 57	13–39
Hypothyroidism (non-Hashimoto's)	28	77	5–35	3.7
Peripheral neuropathy (for example, carpal tunnel syndrome)	28	86	N/A^f^	2.4
Multiple chemical sensitivity syndrome	27	87	4–72%	13%
Sleep apnea	26	65	4–46%	26%
Temporomandibular joint disorder (TMJ)	21	57	27–67%	15%
Postural orthostatic tachycardia syndrome (POTS)	20	83	13–81%	0.17%

a *Subjects were asked if they had ever been diagnosed by a healthcare professional with any of a list of 43 medical/ psychiatric conditions*.

b *Meaning presence of symptoms along with confirmatory bloodwork. Seventy percent stated their mononucleosis was due to Epstein-Barr virus while 16% noted cytomegalovirus-associated mononucleosis*.

c *The most common autoimmune illness was Hashimoto's thyroiditis with a prevalence of 15%. Others mentioned were vitiligo, celiac disease, psoriasis, ulcerative colitis, interstitial cystitis, Sjogren's syndrome, and multiple sclerosis*.

d *Prevalence figures for conditions cited are from the following references: anxiety (24, 39, 41, 51), depression (21, 24, 40, 41), fibromyalgia (19, 24, 26–28, 35, 39, 41, 51, 52), irritable bowel syndrome (19, 25–29, 52), symptomatic infectious mononucleosis (25), migraine headaches (53), autoimmune conditions (25, 40, 54), chronic sinusitis (55), hay fever (55), hypothyroidism (general) (24, 41, 55), multiple chemical sensitivities (19, 27, 35, 55), sleep apnea (41, 56), TMJ (27, 28, 51), POTS (35, 42, 52, 57, 58)*.

e *Prevalence figures for conditions are cited from the following references: anxiety (59), depression (59), fibromyalgia (60), irritable bowel syndrome (61), symptomatic infectious mononucleosis (62), migraine headaches (63), autoimmune conditions (64), chronic sinusitis (65), hay fever (65), hypothyroidism (general) (66), peripheral neuropathy (67), multiple chemical sensitivities (68), sleep apnea (69), TMJ (70), POTS (58)*.

f *No study found addressing this topic specifically*.

## Family Medical History: First Degree Relatives

Thirteen percent recounted at least one first-degree relative (FDR, e.g., blood-related father, mother, sibling, child) who was also diagnosed with ME/CFS and 21%, at least one FDR affected by “chronic fatigue of unclear etiology.” In total, 27% of subjects came from families with FDRs affected by ME/CFS or “chronic fatigue of unclear etiology.” Thirty-five percent of subjects also described at least one FDR afflicted by an autoimmune disorder.

## Medications

Table 7 shows the five most common categories of medications and specific medications taken by this cohort. Medications included prescription and over-the-counter drugs as well as herbal preparations, vitamins and other supplements. Approximately or slightly over 50% of our subjects took a medication regularly or occasionally to manage symptoms related to sleep, pain (not including migraine headaches), and endocrinological issues. Mood and gastrointestinal symptoms were also noteworthy with about one-third of subjects taking medication to cope with these categories of symptoms.

**Table 7:** Most common medication categories and specific medications^a^ used by our subjects^b^.

**Reason for medication**	**Percentage using medication category/specific medication (%)**
Sleep	62
Pain, inflammation, or muscle spasms (not including migraine)	52
Thyroid function, other endocrine/hormonal issues	46
Anxiety, depression, or general mental health	36
Digestive or gastrointestinal problems	35
**SPECIFIC MEDICATION**^**c**^	
Ibuprofen	25
Levothyroxine	20
Melatonin	19
Acetaminophen	17
Zolpidem	15

a *Subjects were initially asked if they took medications for a specific reason (e.g., sleep). Next, they were presented with a list of medications commonly used to treat that condition. A category labeled “other” accompanied by an open text box was also included. Subjects were encouraged to write in anything they took, including herbs, supplements, and over-the-counter medications*.

b *Out of 150 subjects total*.

c *27% and 24% of subjects chose the “Other” category, respectively, for sleep and gastrointestinal treatments. No one medication emerged as dominant. Examples of sleep treatments: over-the-counter pain/ cough medications containing antihistamines; herbal teas; magnesium; L-tryptophan. For gut symptoms: probiotics, prebiotics, digestive enzymes, sodium bicarbonate*.

The most prevalent specific medications matched those of the medication categories. Four of the top five medications addressed sleep (melatonin, zolpidem) or pain (ibuprofen, acetaminophen) while levothyroxine was prescribed presumably for hypothyroidism. Approximately a quarter of our subjects wrote in a medication other than those listed to treat their sleep and gut symptoms. However, no single written-in treatment was used by a significant number of subjects.

## Discussion

This is the first publication to give a broad epidemiologic overview of a US-based, ME/CFS cohort within one paper. While our findings concerning onset, course, function, co-morbid conditions, and personal as well as family medical history are consistent with those of prior studies, we hope to highlight under-examined aspects of this condition: (a) onset is most commonly gradual and precipitated by an infectious incident with stressful/ major life events as the next most frequent precipitant; (b) problems with prolonged standing, alcohol consumption, and temperature regulation, which all may be related to circulatory impairment, are common; (c) while other symptoms may decline over time, cognitive symptoms tend to persist; (d) improvement in our cohort is rare but short, temporary remissions can occur in a minority of patients; (e) increasing age and illness duration do not necessarily portend worsening fatigue or function; (f) events associated with the female reproductive system can negatively impact ME/CFS in women; (g) patients with co-morbid medical or psychiatric conditions are the rule rather than the exception; and (h) ME/CFS, chronic fatigue of unclear etiology, and autoimmune conditions are common in family members. These findings have significant implications for the clinical care and research of patients affected by ME/CFS. We also supply information about medication usage for context.

## A Gradual Onset Preceded by an Infectious Event is the Most Common Pattern

The most common onset pattern was a distinct change in health heralded by an infectious event followed by a gradual progression to becoming consistently sick. Despite offering 14 possible precipitating factors and an open text box, almost two-thirds of our subjects selected only one or two factors. The top three factors were an infectious illness (64%), stress or a major life event (39%, e.g., occupational pressure, family illness, divorce), and exposure to an environmental/ chemical toxin (20%, with mold being the top answer written in) (Table 1). Stressful events were rarely chosen as the only precipitant though, endorsed only by 8% of our subjects, and appeared mostly in conjunction with infection or other precipitants. These results agree with prior studies: 49–93% of subjects reported an infection-like illness while 43–95% noted significant stress in the months or years preceding or surrounding the beginning of their illness (18, 41, 42, 52, 71–74). Becker (72) also found that 99% of their subjects chose only 1 or 2 factors and both he and Evans (52) showed <15% of subjects endorsed stress as the sole precipitant.

Since infectious events have been consistently found to be the foremost factor preceding ME/CFS retrospectively and prospective studies (14, 15) have confirm their progression to ME/CFS, this fact should be emphasized more in educational materials and case definitions. There are already two moves in this direction. The 2015 National Academy of Medicine criteria, also known as Systemic Exertion Intolerance Disease (SEID), incorporated failure to recover from infection as a secondary characteristic of ME/CFS (75) but perhaps this characteristic should be elevated to primary criteria. Lack of or incomplete recovery might provide a valuable clue in the diagnosis of ME/CFS for clinicians faced with a plethora of patients presenting with fatigue. Additionally, the National Institutes of Health have focused on post-infectious cases of ME/CFS in their intramural ME/CFS study (76) to try to decrease the heterogeneity of their research sample. Heterogeneity of study samples is widely acknowledged to be an obstacle for ME/CFS studies (77). As shown in Table 1, we believe that other precipitating factors should be explored. Nevertheless, materials which do not emphasize a post-infectious onset (78) or suggest that all precipitating events are equally common or relevant (79) make it more difficult for clinicians and researchers to discern ME/CFS from other medical conditions or situations.

For a third of subjects each, their preceding infectious event manifested as respiratory symptoms (e.g. sore throat, rhinorrhea, cough, etc.) or constitutional symptoms (e.g., fever, chills, muscle aches, etc.) while 35% claimed that a specific infection was documented (Table 2). This is comparable to Ramsey's early accounts (80) and Becker's (72) study where upper respiratory infections (URIs) were noticed to be the chief infectious event, followed by “flu-like illness[es],” and, trailing far behind, gastrointestinal infections. In our survey, gastrointestinal (GI) infections were also rare, endorsed only by 10% of those with an infection peri-onset and 6% overall (out of 150 subjects). This might clue clinicians in to a diagnosis of ME/CFS even without a specific pinpointed microbe as certain types of infections (e.g., prostatitis, urinary tract infections, etc.) seem much less likely to progress to ME/CFS.

However, respiratory symptoms should not lead researchers to assume that the common causes of URIs (e.g., rhinoviruses, coronaviruses) are implicated in ME/CFS. In 1998, White (81) demonstrated that URIs are much less likely to cause ME/CFS than glandular fever. The pathogens which have been linked to ME/CFS are extraordinarily adept at evading the usual immune defenses through, for example, intracellular or latent states (e.g., Coxiella burnetii, herpes family viruses). Thirty-five percent of our subjects noted documentation of an acute infection, higher than the 23% Salit (71) found. Unfortunately, our survey did not ask respondents to elaborate which initial infections they were diagnosed with, a shortcoming we hope to rectify in the future. Knowledge of which pathogens contribute to the establishment of ME/CFS may stimulate new ideas about disease pathophysiology and treatment.

The frequent presence of stressful/ major life events surrounding ME/CFS onset does not automatically mean that ME/CFS is caused by or perpetuated by psychiatric or psychological factors. Some researchers have proposed or supported psychogenic or psychosomatically-infused theories of ME/CFS which have led to therapies like cognitive behavioral therapy (CBT) and graded exercise therapy (GET). These treatments are predicated on the hypothesis that patients are overly anxious or fearful about normal bodily sensations *per se* or minor discomfort related to deconditioning and thus limit their activities (82). Both treatments have been shown to not only be much less effective than initially alleged (83) but to actively cause harm to patients (84). Metabolic studies provide objective evidence that patients' bodies are unable to routinely meet energy demands (85).

Other mechanisms may be responsible for the recurring association between stressful/ major life events and ME/CFS. One theory is that stress decreases the immune system's ability to fight off and contain infections (86). Some ME/CFS patients are significantly helped by administration of antivirals (87, 88) while other studies suggest deficient control of infections (89, 90). Alternatively, peri-onset stressful events might act as the “straw that broke the camel's back,” accelerating a pathological process which was already underway. A hallmark feature of ME/CFS is post-exertional malaise (PEM), a severe and often prolonged exacerbation of multiple symptoms (e.g., muscle pain, fatigue, problems thinking, sore throat) which is out-of-proportion to the physical, cognitive, emotional, or positional stressors triggering it (6, 44, 91). PEM can be triggered by activities of daily life (e.g., showering, cooking, reading, etc.) and is often deemed to be the most disabling ME/CFS symptom. Several studies provide evidence that ME/CFS patients' bodies react to these stressors abnormally. For example, compared to healthy people, the rise of serum cortisol and heart rate in response to, respectively, the stress of waking up (92) and aerobic exercise (93), are blunted in ME/CFS patients. Conversely, when the sympathetic nervous system involved in reacting to stress should be dampened, for example, during nighttime to facilitate sleep, its activity is instead elevated, possibly leading to another major ME/CFS symptoms, unrefreshing sleep (56, 94). Combining what is known about onset with these key symptoms suggests that a dysfunctional stress response system may play a major role in the pathophysiology of ME/CFS.

The stress response system in the human body depends on a complex interplay between the neurological, neuroendocrine, and endocrine systems (95, 96). Components involved in the response system include the prefrontal cortex, amygdala, hypothalamus, pituitary gland, sympathetic and parasympathetic ganglia and nerves composing the autonomic nervous system, and adrenal glands. The theory behind CBT depends on defects at the beginning of this system, i.e., the cognitive appraisal and interpretation of challenges. Consequently, it is believed if patients can decrease their fear and anxiety around activity and symptoms then their ME/CFS will be largely resolved. However, problems with any downstream component of the stress response system could also lead to poor adaptation and similar consequences. Autoantibodies to both adrenergic and cholinergic muscarinic receptors (97, 98), part of the signaling pathways in the sympathetic and parasympathetic nervous systems, have been found in a subset of ME/CFS patients. They may account for some patients' bodies reacting inappropriately to stressors. Other individuals' symptoms might instead stem from other components, like the hypothalamus or adrenal glands. Different, dysfunctional components of the stress response system may potentially generate different clinical presentations while still preserving the hallmark feature of PEM.

The aforementioned concepts are not entirely new. In 1995, to explain why 95% of their ME/CFS subjects endorsed a stressful event at onset, Dobbins et al. (73) initially advanced stress as a causative factor or as a byproduct of recall bias but then also considered that “the perception of stress is [also possibly] correlated with some other variable related to the pathogenesis of CFS.” These concepts are testable. By prospectively following adolescents stricken by Epstein-Barr mononucleosis, Katz et al. (99) have already demonstrated that autonomic dysfunction is predictive of ME/CFS several months later. Future studies could attempt to replicate Katz's study, especially in adults, after different types of infections known to be linked to ME/CFS, and in parallel with both subjective measures of challenges (whether physical, cognitive, emotional, or orthostatic) and objective measures of the stress response system (e.g., tilt table, serum cortisol levels, thermoregulatory sweat test, heart rate response to Valsalva maneuver).

Ideas about acuity and its link to infection should also be re-examined. Some past case definitions have included onset within a few hours or days as part of their criteria (100–102). In contrast, for the majority of our subjects, the first intimation of illness to full-blown ME/CFS often occurred over months if not years (Table 3). This is congruent with empirical data: while a few studies reported that around 60% and up to 91% of subjects disclose an “acute” onset (42, 52, 72), the majority of subjects (between 59 and 77%) in many studies describe a “gradual” onset. (37–39, 41, 103). Furthermore, many researchers do not define, are vague, are or inconsistent among themselves about what period of time (e.g., hours, days, weeks) is considered “acute.” When interviewed in detail by Evans et al. subjects choosing a “sudden onset” described time periods ranging from a few hours to a few years and interpreted the term to mean remembering a discrete onset date, experiencing a severe onset, or having an infection around the time of ME/CFS onset (52).

We also found that there was no link between subject endorsement of an infectious precipitant and the time span of ME/CFS development. Some believe that an acute onset is necessarily infectious or an infectious onset is necessarily acute (50). Past studies examining this relationship are mixed, with some agreeing (52, 72) and others disagreeing with our result (104). Clinically, one infectious yet gradual onset sequence we have observed is a stuttering pattern whereby a subject experiences a severe infection, returns to near-normal functioning, but then experiences recurrent infections over months to years, recovering less each time, before succumbing entirely to ME/CFS. Overall, we agree with Evans that onset patterns are complicated and that simple categories do not capture this complexity. In the meantime, researchers should be careful about mandating an acute onset in order for an individual to be diagnosed with ME/CFS and should not make assumptions about the relationship between duration of onset and etiology. Future studies need to be more precise about what they are studying: if it is about time, define the time periods; if it is about infection, ask about infection. Accurate representation of onset is important as it might provide the key to the pathophysiology of ME/CFS.

## Further Exploration of Other Potential Triggers is Needed

Twenty percent of our subjects noted that an exposure to a chemical or environmental toxin might have a played an initiating role in their illness. Two independent Australian research groups published similar results: in Clark et al. (41), 16% endorsed “exposure to environmental toxins” while in Johnston et al. 6% reported “mold”; 11%,“toxic chemicals”; 6%, “poor[ly] recycled air”; and 4%, “heavy metals” (42). In contrast, in Friedberg's US-based study, 44% of subjects perceived “toxic exposure” to be a source of their illness (18). It is unclear why Friedberg's study yielded double the percentages we and the Australians found. None of these researchers commented further on these findings in their articles.

Since this survey was constructed as a broad overview, we did not take a comprehensive history of possible exposures. Additionally, because the published literature in this area is sparse and composed of mostly case studies [e.g., (105, 106)], it is difficult to know what specific substances to concentrate on. Written-in responses from our subjects may provide leads but were too imprecise and disparate to draw any solid conclusions. Subjects' answers might also be influenced by, for example, recall bias, misattribution, other patients' accounts, and media outlets.

Nevertheless, given how often this topic has come up, it is an area deserving of more attention. One initial approach might be to formally survey clinicians about what external, non-infectious triggers they believe to be important. Patients could be asked if anyone around them suffers from similar symptoms and if there are places where or times (of the day, week, or year) when they recurrently feel better. These traits have been suggestive of an environmental factor in other medical conditions. If patients respond affirmatively, clinicians should take a more detailed occupational, residential, and avocational history. Establishing a causal link between a particular agent and a disease is challenging. Definitive answers are often impossible to obtain although well-designed toxicological and epidemiological studies performed in parallel can reach sensible conclusions (107).

We encountered similar issues with replies to our items regarding travel. Patients have occasionally stated that they became ill during or shortly after a trip or that they have a history of widespread travel. Many wonder whether their excursions have any relationship to their illness. Responses collected in this survey were too diverse to generate concrete hypotheses. It is also plausible that the unpredictability and hassles of travel itself (i.e., stress) instead of subjects' destinations were conducive to illness onset.

## Alcohol Intolerance, Thermoregulation, and Difficulties Standing Still are Common Symptoms

Our subjects confirmed the high frequency of symptoms often considered important features of ME/CFS by clinicians but not included in the 1994 Fukuda CFS criteria. The prevalence of alcohol intolerance (66%), difficulties managing temperature extremes (87%), and issues with standing (81%) are as high or higher than some of the top 12 symptoms in our Table 4. Additionally, they are within the range of prevalence figures found previously (Table 8): 45–80% for alcohol intolerance (57, 108–110); 54–80% for temperature control issues (16, 108, 109); and 81–95% for problems with remaining immobile in an upright position (111–113). Bansal has suggested that since alcohol intolerance is present in 80% of his ME/CFS patients, its occurrence should increase the likelihood of an ME/CFS diagnosis if there are any doubts otherwise (110). Based on his finding that 81% of ME/CFS patients demonstrated abnormal tilt table testing results, Lapp proposed that all patients should be questioned about orthostatic intolerance (111). The symptom most predictive of an abnormal test was not fainting/ near-fainting but inability to stand in place without getting sick.

**Table 8:** Prevalence of self-reported alcohol intolerance, thermoregulatory issues, and difficulty standing still in this and other studies.

**Symptom**	**Prevalence in subjects with ME/CFS (%)**	**Study author (references number)**
Alcohol intolerance	66	This study
	45–75	Berne (108)
	60	De Becker (109)
	67	Woolley et al. (57)
	80	Bansal (110)
Problems adjusting to heat or cold	87	This study
	59	Chu et al. (16)
	75–80	Berne (108)
	54	De Becker et al. (109)
Difficulty with standing still^a^	81	This study
	81	Lapp et al. (111)
	95	Rowe et al. (112)
	90^b^	Robinson et al. (113)

a *Due to symptoms associated with orthostatic intolerance*.

b *Composite of symptoms including orthostatic intolerance*.

Dysfunction of the autonomic nervous system is one mechanism which may account for all three non-Fukuda symptoms. Without appropriate vasoconstriction and vasodilation of blood vessels by the autonomic nervous system, consistent blood pressure and body temperature may not be maintained, resulting in postural and thermoregulatory issues (114). Alcohol not only increases fatigue and disturbs cognition but also has been shown to exacerbate orthostatic intolerance (115), compatible with why some patients affected by ME/CFS would endorse problems with alcohol intake.

Currently, OI is already one of five symptoms highlighted by the NAM (6, 75) but intolerance to climatic shift is not. Both symptoms are included in the CCC (49) and ME-ICC (50) but buried in a long list of other symptoms and are optional. Emphasizing these symptoms would not be a new undertaking but actually a return to Dr. Melvin Ramsey's original conception of ME where “circulatory impairment” manifested as “hypersensitivity to climatic change,” insufficient responses to stress, and “dysfunction of the autonomic nervous system” (80) are repeatedly mentioned. These symptoms could be especially selected for when recruiting subjects so that they may be investigated further.

## Disease Course

Over time, while individual symptoms or the disease overall might fluctuate and even remit temporarily, almost all of our subjects continued to be sick and disabled. During the first 6 months of the illness (Table 4), the most common symptoms were fatigue-, exertion-, sleep-, pain-, cognition- and flu-related, with over 70% of subjects endorsing these symptoms. Similarly, Evans found that exhaustion (57%), cognition (43%), headaches, pain, and sleep were the top symptoms at onset (52). As months and years passed, most symptoms remained among the top 12 most common symptoms even as the percentage of subjects experiencing any symptom declined. Feeling fatigued and unrefreshed after a night's sleep retained their ranking as the most and second most common symptoms. Because the declines associated with troubles paying attention, finding the right word and remembering things were relatively small (between 4 and 10%) compared with those of flu-like symptoms, dead/heavy feelings post-exercise, and muscle pain/ aches (between 17 and 25%), these cognitive symptoms rose in their ranking while the latter three fell off the most common dozen symptoms. Similarly, because of their low declines in prevalence (between 1 and 10%), three other cognitive symptoms (absentmindedness, inability to multi-task, and sensitivity to noise) joined the top 12 symptoms by the time of the survey.

Our results agree with two published studies examining symptoms in subjects who remained sick for over a decade. Sore throat and lymph nodes tenderness tended to improve the most over a mean of 15.4 years of illness (24). On the other hand, Friedberg noted that the third, fourth, and fifth most common symptoms in subjects sick for a median of 18 years were “forgetfulness,” “distractibility by noise,” and “concentration difficulty” (18). Additionally, when Friedberg analyzed which symptoms were significantly more frequent in these long-term subjects vs. his short-term subjects (median length of illness = 3 years), four out of the top five symptoms were cognitive symptoms. After a median of 12.5 years of sickness, we observed remarkably similar shifts in our study sample. Inability to multi-task rose from being the 22nd most common symptom to 8th most common; forgetfulness from 19th to 10th; and nose sensitivity from 15 to 12th. In contrast, Jason (19) found little change in prevalence when comparing Fukuda-associated symptoms assessed at two time points separated by a decade. Discrepancies in findings might be traced back to the cross-sectional vs. longitudinal design of studies, how subjects were selected, whether symptoms were inquired prospectively or retrospectively, varying follow-up times and the stage of subjects' illness when they were questioned. Ideally, research investigating evolution of symptoms should be prospective, longitudinal, and endure beyond a few years.

The reasons why symptoms fluctuated differed according to the individual symptom. Patients recounted that fatigue, post-exertional malaise, and unrefreshing sleep appear to be improved by treatment whereas flu-like symptoms abated spontaneously. This latter claim is supported by Lipkin et al. (116), who found that subjects ill for <3 years demonstrated more robust pro- and anti-inflammatory activity relative to subjects who had been ill longer. We did not ask patients specifically which treatments helped the most but use of a behavioral technique called pacing along with sleep medication are often deemed to be helpful among patients (16, 117). The stubborn presence of cognitive symptoms is concerning. Clinical trials targeting the cognitive symptoms of ME/CFS or including neurocognitive outcome measures are rare: both deficiencies need to be remedied.

The dominance of infections and stressful/ major life events as significant modifiers of disease course underscores the importance of these two factors (Table 5). The third most common answer, “None of the above,” was selected by a quarter of subjects and all other choices were selected by 11% or less of subjects. Intervening medical events (e.g., surgery, accidents, cardiac and neurologic disease) also, unsurprisingly, impacted the overall course of the disease. This result concurs with March et al. and others (24, 117, 118) who have shown that additional co-morbidities tended to worsen ME/CFS symptoms and function. The lack of long-term longitudinal studies means there is very little information about what issues or events influence disease course. Finding out more about this area may aid in understanding ME/CFS and bring up opportunities for secondary prevention (e.g., decrease functional decline). For example, Dr. Charles Lapp (119) has written previously on steps clinicians can take to prepare patients for and minimize the effects of surgery.

Surprisingly, about one-tenth of our subjects experienced complete cessation of their symptoms during their illness course even as their ME/CFS eventually recurred. Similarly, over a short follow-up period of 3 years, Nisenbaum et al. (21) found that about 10% of their subjects sustained “total” remission of at least a year's time. However, since remission was defined by operational criteria rather than direct questioning of their subjects, the authors believed that actual remission rates might be lower. In March's study (24) of long-term ME/CFS subjects, the prevalence of any remission was higher at 30% but they did not specific for how long symptoms were absent. While our median length of remission was 7 months, one subject noted normal health for a decade. This is not without precedent: online anecdotes (120, 121) support long intervening periods of good health between episodes of ME/CFS. These findings accentuate the importance of appropriate control subjects and extended follow-up times. Temporary disappearance of ME/CFS symptoms may confound the interpretation of interventional, longitudinal, and prognostic studies. With a few exceptions, most studies have lasted for <5 years when it is extremely common for study subjects to have been sick for more than a decade. Control subjects and protracted monitoring would help distinguish transient variations from long-term, lasting improvement.

Since the GEISD study was not set up to particularly assess prognosis, we cannot calculate a rate of recovery but the unrelenting illness course of our subjects is consistent with other studies. Only 4% of our subjects felt their medical condition was improving over time with 50% endorsing a fluctuating course. A 2013 survey (16) of 551 subjects found only 1.1% felt they were improving while 54.4% designated their course as “fluctuating/ remitting/ relapsing” and 27%, as “worsening.” Out of 14 subjects she interviewed in-depth, Evans et al. (52) found only 1 (7%) expressing continual improvement while Underhill's rate was 5% (35). These data comport with the low median recovery rate of 5% Cairns (12) found in their 2005 systematic review of prognosis. In individual studies, higher rates of recovery, up to 66%, have been documented but Friedberg et al. (122) as well as Jason (123) have questioned the validity of such figures since recovery definitions have tended to be limited, narrow, and/or unidimensional.

## Events Associated With the Female Reproductive System Affect ME/CFS

Considering that ~75% of people affected are women (16, 40, 41, 43, 104, 124–126) and that onset often occurs during their reproductive years, i.e., between the ages of 10–40 (16, 23, 39–42), exceptionally few studies have evaluated the impact of female reproductive events on ME/CFS. During casual conversations or in the clinic, patients will occasionally relate that their ME/CFS began during or shortly after pregnancy. In one study of stressful events surrounding onset, women who were pregnant in the previous year were found to be 31.7 times more likely (126) to become ill with ME/CFS compared to women who had not been pregnant. A small but detectable 8% of our subjects (Table 1) connected their illness onset to pregnancy, within the range of 3.5–10% identifying this as an initiating event in earlier studies [18. 52]. When the more ambiguous category “hormonal events” was used instead, this percentage rose slightly to 12% (18, 41).

Although women have discussed amongst themselves premenstrual aggravation of their ME/CFS symptoms for many years, only one other study besides ours has formally surveyed patients. Sixty-seven percent of Clark et al.'s (41) subjects reported worsening of ME/CFS before their periods, close to our figure of 53% (Figure 1). Likewise, there have been scant studies of ME/CFS symptoms during pregnancy. Schacterle (127) showed that approximately equivalent percentages of patients reported no change, deterioration, or a boost in their health status (41, 30, 29%, respectively) during pregnancy, convergent with our figures of 31, 27, and 42%. Conversely, 86% of one Australian cohort (41) reported deterioration while the impression of several US-based ME/CFS specialists (128) was that ME/CFS symptoms tended to attenuate during pregnancy, to the point of remission. One reason for these diverse conclusions might be that ME/CFS symptoms vary depending on the stage of the pregnancy (e.g., first trimester, second trimester, etc.). Without explicit questioning, some subjects might be communicating their average health status during pregnancy while others might inadvertently be focusing on one time period to the exclusion of others.

Despite the highest prevalence of ME/CFS being recorded in the 40–50 age range (124, 129), no other study has asked about the impact of menopause on symptoms. Menopausal symptoms such as increased fatigue, hot flashes, insomnia, and forgetfulness overlap with those of ME/CFS. This fact combined with the 38% of our peri- and post-menopausal subjects (Figure 1) who felt that menopause exacerbated their ME/CFS should prompt further research. Are amplified symptoms during this life phase due primarily to the expected changes of menopause, hormonal adjustments on ME/CFS, or a combination of the two? Should ME/CFS be a consideration when women decide whether and for how long to partake of hormone replacement therapy? In contrast, over three-quarters of women expressed no changes in symptoms while taking exogenous female hormones, whether for birth control, menopause, or other medical conditions. Only eleven percent of our subjects and 7–9% of Friedberg's (18) subjects noted worsening or onset, respectively, with hormonal medications.

Investigating these topics can shed light on the pathophysiology of the disease, answer women's questions about ME/CFS during different stages of their lives, and even result in new treatments. The oscillation of symptoms with these short-term and even repetitive events can provide a naturalistic model for understanding the relationship between biological indices and clinical characteristics. Remissions and flares of various autoimmune diseases during pregnancy have been linked, respectively, to a TH1 or TH2-dominant immunological status (130). Equipped with more knowledge, healthcare professionals can better assist women to make informed decisions about pregnancy and to prepare for menstrual cycles and menopause. New treatment options might even be introduced. For example, anecdotal evidence (131, 132) suggests that some women may be able to moderate their premenstrual intensification of ME/CFS symptoms with judicious use of birth control pills or patches. These management techniques need to be tested in formal clinical trials.

## Function is Low But Appears Stable Over Time

The high rate of unemployment we observed (47%) is in line with the 40–81% rate noted in other studies (7, 16, 21, 23, 24, 39, 41–43, 133). Commencement of ME/CFS decimated the pre-illness employment rate by at least 40% in Japan (43), Australia (41), and the United Kingdom (133). Moreover, surveys rarely asked those still employed if they were able to retain their prior hours, duties, position, salary, or even field: in the Japanese study, only 2% of respondents did not have to modify their occupation whereas both Tiersky (20) and Kingdon (133) found much reduced work hours. Functional levels echoed those of a 2013 survey of over 550 subjects (16): even during their best periods, most subjects could barely attend to school, work, or family responsibilities part-time (but not all three) and during their worst periods, over half were bedridden and unable to participate in any activities (Figure 2).

These low functional levels are supported by the high mean FSS and MFI-20 scores [respectively, 5.9 ± 1.1 (out of 7) and, for example, MFI General Fatigue (GF) subscale 17.2 ± 3.0 (maximum of 20)], which reflect those of prior studies (134–137), are occasionally double the score of healthy controls, and even exceed the mean values of subjects afflicted by depression, stroke, multiple sclerosis, myocardial infarct, systemic sclerosis, and human immunodeficiency virus (134, 136–140). Of the studies examined for comparison, only patients affected by fibromyalgia/ chronic widespread pain (138) or enrolled in palliative care programs for cancer (140) exhibited mean MFI-20 GF scores (respectively, 16 ± 3.2 and 17 ± 3.0) approaching those of ME/CFS subjects.

Despite how severely ME/CFS impaired our subjects, it may be reassuring to clinicians and patients that functional status does not seem to drop with the passage of time. As shown in Figures 3, 4, no relationship was observed between either measure of fatigue and age of subjects or duration of illness. These results agree with studies monitoring Short Form 36 physical function (SF-36 PF) subscale trends across time. In a cross-sectional survey of ~500 subjects, Chu et al. (141) found low, non-significant Pearson correlation coefficients of 0.025 and 0.019 when SF-36 PF scores were plotted against age and duration of illness. In fact, Komaroff (142) found slight improvements in SF-36 PF when following one cohort of 99 subjects over a decade and both Tiersky (20) and Kidd (125) mentioned that long-suffering subjects might develop better psychological coping techniques. One study (143) did indicate increased fatigue, autonomic symptoms and depression in older subjects relative to younger ones but it is unclear how this study's conclusion might apply to the question at hand since they emanate from a sample who did not develop ME/CFS until they were 55 years of age or older, 15–20 years beyond the mean age of ME/CFS onset. Indeed, the authors speculated that ME/CFS beginning in later stages of life might be very different from earlier-onset ME/CFS.

Our results should be interpreted with caution since a) they derive from a cross-sectional instead of longitudinal cohort and b) the FSS is subject to ceiling effects (144). Since cross-sectional designs are based on different subjects, it might not be accurate to extrapolate future function from one individual to another. Because two-thirds of our subjects displayed an average score of 6 or higher on the FSS when 7 is the maximum score, the FSS might not have the capacity to represent or distinguish between more intense levels of fatigue.

## Multiple Co-morbid Conditions are the Rule Rather than the Exception

ME/CFS is often accompanied by other co-morbid and psychiatric conditions. Out of 43 listed conditions, almost all our subjects (97%) had been diagnosed with at least one medical condition while 64% revealed at least one psychiatric condition. The mean number of conditions affecting subjects was high, 7.0 ± 4.2. Previously, in separate studies, 80–95% of subjects have declared at least one other condition while 38–90% cited at least one psychiatric condition (19, 24, 28, 29, 39, 51, 52, 105). In Bateman et al.'s study (29), out of 17 conditions listed, women subjects suffered a mean of 2.7 ± 2.1 conditions and men, 3.6 ± 2.1 conditions.

Our 5 most common conditions (anxiety, depression, fibromyalgia, irritable bowel syndrome, and migraine, from most to least common) (Table 6) match 3 of the top 5 condition in Bateman's cohort (fibromyalgia, depression, anxiety, low testosterone, hypothyroidism) (29). We did not ask about low testosterone, which existed in 36.4% of their male subjects, and hypothyroidism was our ninth most common condition albeit our prevalence of 28% is close to their 35%. Their survey did not query about irritable bowel syndrome or migraine headaches.

Our prevalences for individual conditions are generally concordant with those documented in previous ME/CFS studies (Table 6). The exceptions in this comparison are migraine headaches (37% in our study vs. 84%) (53) and chronic sinusitis (33 vs. 66%) (55). As there is meager data on these two conditions in ME/CFS however, these comparisons should not be taken as the final word. In contrast, except for sleep apnea and symptomatic infectious mononucleosis, all 12 of the most common conditions in our study surpassed their prevalence in the general United States population [Table 6, (58–70)]. The similar prevalence of mononucleosis in our subjects compared to the general population [37 vs. 30–50%, (62)] suggests that mere symptomatic infection does not elevate the risk of ME/CFS but rather the severity or aftermath of the infection may be the determining factor for whether ME/CFS manifests. Intriguingly, our prevalence of cancer (8%) is approximately double that of the prevalence in the general population [4.1%, ages 50–59, 14-yr. limited prevalence, (145)], whereas Bateman's cohort had quadruple the prevalence at 16% (29). These results, along with studies showing an increased risk of lymphoma among elderly ME/CFS subjects (146) and an early mean age of death due to cancer (147), warrant further investigation.

Our data underscore the importance of the National Academy of Medicine criteria (6) moving away from ME/CFS being primarily a diagnosis of exclusion and allowing the concurrence of what some clinicians and researchers might have interpreted to be absolute exclusionary diagnoses (e.g., major depression, obstructive sleep apnea, hypothyroidism). Given the ubiquity of co-morbid conditions, many ME/CFS patients might never be diagnosed with or would have lost their ME/CFS diagnosis had the NAM criteria continued to designate exclusionary criteria. Some people (148) have expressed concerns about how the new criteria might unintentionally attract subjects to studies who are actually affected by another diagnosis or have a potentially confounding condition (e.g., major depressive disorder). However, depending on a study's purpose, researchers can always institute additional exclusionary criteria beyond the NAM criteria or alternatively, subgroup or statistically adjust for co-morbid conditions. The internal and external validity of a study must also be balanced (149): strict exclusionary criteria might permit more solid conclusions to be made but the results might not have much applicability for the average ME/CFS patient (150).

Given their unresolved/ active state, the most common co-morbid diagnoses should be actively screened for by healthcare professionals. Treatment options for ME/CFS itself are limited but many of these conditions have standardized, effective treatments. Their improvement can positively influence patients' health, function, and quality of life even as they remain ill with ME/CFS (117, 118). Finally, studying co-morbid conditions might also help provide answers to the pathophysiology of ME/CFS. For example, the high prevalence of autoimmune co-morbid conditions supports the hypothesis that ME/CFS might be an autoimmune condition for at least some subgroups or that the immune system possibly plays a major role in disease pathophysiology. On the other hand, the unexpected high prevalence of migraines reinforces the idea of ME/CFS being a condition of poor autonomic dysfunction. Some (151) have postulated that imbalance of the sympathetic and parasympathetic arms and changes in cranial blood vessel dilation are two of the many steps leading to a migraine headache.

Aside from natural variation in study samples, the wide range of prevalence for the same co-morbid condition could be due to issues like which conditions clinicians were alert to, how study subjects were asked about medical conditions, the accuracy of subject recall, and how subjects were assessed for a co-morbidity. For example, March did not ask about co-morbid conditions overall but only about conditions after onset of ME/CFS. Conditions which are less recognized by clinicians will also be less likely to be diagnosed: this might account for why only 13–40% of subjects stated they were diagnosed with POTS (Table 6) whereas unfiltered screening of all ME/CFS patients in Lapp's study (111) yielded an 81% prevalence of POTS.

## ME/CFS, Chronic Fatigue of Unclear Etiology, and Autoimmune Disease are Common in First-degree Relatives

The pervasiveness of ME/CFS, chronic fatigue of unclear etiology, and autoimmune disease in the FDRs of subjects may yield clues to the genetic basis and pathophysiology of the disease. Thirteen percent of our subjects imparted that they had at least one FDR affected by ME/CFS. Because ME/CFS is known to be widely underdiagnosed (152–154), we also asked whether any FDRs sustained chronic fatigue without a specific diagnosis. When this category was added, 21% of our subjects replied affirmatively and the percentage of subjects who did or might have a relative afflicted by ME/CFS rose to 27%. Our results are consistent with Pheby (34), who found that 12.1% of his subjects had at least one FDR affected, and the 5.3–18.3% of subjects noted in multiple studies (30, 32, 35, 36, 41, 155) to have at least one other blood-related family member, regardless of degree, affected. In two studies asking about both ME/CFS and chronic fatigue of unclear etiology in family members, 25% (31) and 46% (35) replied affirmatively.

ME/CFS has also been shown to be present in second- and third-degree relatives (33, 35) in a dose-response matter, i.e., the more genetic distance between the ME/CFS patient and a relative, the lower the risk. Since second- and third-degree relatives are less likely to share the same household or lifestyle factors as FDRs, this pattern strengthens the argument that there might be a shared genetic rather than environmental factor increasing the risk of disease. Astonishingly, hardly any studies have examined families where multiple members are sick with ME/CFS. More than 2 decades ago, Levine (156) showed a gradient of natural killer cell activity with family members affected by ME/CFS having the lowest values, followed by those unaffected but related, and finally, un-related friends of the family having the highest and normal values. By evaluating such family pedigrees, especially in conjunction with genetic or other biomarkers, we might better comprehend the risk factors behind and the mechanisms of ME/CFS.

About one-third of our subjects suffered from an autoimmune condition (Table 6) and a similar percentage had an FDR with an autoimmune condition. These figures are congruent with prior research: autoimmune conditions were noted in 15–27% (40, 54) of ME/CFS patients and in 18–47% (40, 54, 155) of their family members. Autoimmune thyroid disease was the most common co-morbid diagnosis while a variety of conditions, including rheumatoid arthritis, psoriasis, lupus, and Sjogren's syndrome, were observed among family members.

The commonality of autoimmune conditions within patients and among their family members is compatible with some researchers' theories (157, 158) that ME/CFS might have an autoimmune basis. It is well-known that individuals with one autoimmune disease are more likely to be affected by another autoimmune disease (159). The same but also diverse autoimmune diseases might affect families; the former phenomenon is labeled as a “familial autoimmune disease” while the latter is known as “familial autoimmunity” (160). Furthermore, many of the traits displayed by ME/CFS fit Rose and Witebsky's circumstantial criteria (161, 162) for determining when a condition qualifies as an autoimmune condition. For example, ME/CFS is more common in women, runs in families, can be triggered by infections, can be alleviated by immunosuppressants and is associated with autoantibodies [e.g., to adrenergic and cholinergic receptors (97, 98)]. Fluge et al. (163) also demonstrated *in vitro* that serum transferred from patients' bodies adversely affected the function of healthy, cultured muscle cells. This serves as a more direct piece of evidence for autoimmunity. Rose and Witebsky's criteria could operate as a guideline for future studies to prove or disprove the role of autoimmunity in ME/CFS. For example, to test maternal transfer of autoantibodies, infants of ME/CFS patients could have their blood tested for ME/CFS-specific autoantibodies and be followed serologically and clinically for ME/CFS symptoms. Four percent of our subjects and 6% of Jason's (164) admit to being sick as long as they can remember. Another project might devise animal models capable of developing ME/CFS: if exposure to patient serum or a putative antigen replicates the illness in these animals, that would corroborate the autoimmune foundations of ME/CFS.

## Medications

Unsurprisingly, the most common specific medications and categories of medications used (Table 7) correspond to well-known ME/CFS symptoms (sleep, muscle/ joint pain, headaches) and co-morbid conditions (mood disorders, fibromyalgia, irritable bowel syndrome, hypothyroidism). Our findings are similar to the medication survey which Reeves et al. (165) conducted in 2003. Their top six medication categories were pain relievers (88%), hormones (52%), antidepressants (41%), allergy-related drugs (32%), gastrointestinal therapies (30%), and cold medications (25%). Sixty-two percent of their subjects took supplements and vitamins.

Reeves did not ask subjects why they used specific medications and attributed allergy-related/ cold medications to the alleviation of sore throats, which are part of ME/CFS. While it also possible subjects are taking these medications for hay fever or sinusitis (see Table 6), subjects may also be consuming antihistamines to assist with sleep. (Reeves et al. classified antihistamines as both allergy and cold medications.) This claim is reinforced by patient comments from a survey conducted for a US Food and Drug Administration workshop in 2013 (16).

The pervasiveness of over-the-counter medications, herbal preparations, and supplements underscores the need for research directed at symptom control. For example, Gotts et al. have mentioned targeting the different phenotypes of sleep issues in ME/CFS with different medications (166). Subjects also expressed that side effects or hypersensitivity to customary doses of medications restricted what they could use. Effective management of symptoms can help patients greatly while progress is being made toward a disease-modifying treatment.

## Strengths and Limitations

The major strength of this study lies in its use of a collaboratively-designed survey covering a broad range of topics relevant to clinicians, researchers, and patients. Consequently, except for a few items, the overall amount of missing data was small. Because data were collected from a single cohort, we were able to make connections between different areas (e.g., onset time and infectious onset) and confirm that, despite claims that ME/CFS is a heterogenous disease, separate aspects of epidemiological information collected from many cohorts based in different locations and at different times are in concordance with the data captured from one cohort. Our results also contribute to the paucity of data on the evolution of symptoms longitudinally and the impact of female reproductive events on ME/CFS.

Limitations of this study include the study sample recruited, reliance on subject self-report, recall bias, and relative superficiality of some survey items. Although we tried to recruit subjects from a diverse range of sources, our study population still consisted primarily of middle-aged, self-identified Caucasian women who had been sick for over a decade. Most ME/CFS studies end up with a similar sample. Therefore, the results of our study may be less applicable to younger, male, non-Caucasian, and recently afflicted patients. During the recruitment stage of this study, in 2012, the prevailing research case definition was the Fukuda 1994 criteria. In 2017, the US National Institutes of Health announced that either the CCC or NAM criteria should be used instead (167). Despite this study's use of the Fukuda 1994 criteria, we believe that our results will also apply to subjects fitting CCC or NAM criteria: at least 71 and 72% of our subjects qualified, respectively, for these criteria.

Because items answers were based on subject self-report instead of medical records or clinical examinations, some portions of the survey (e.g., peri-onset factors, comorbid conditions) might be less accurate than others. Moreover, since our subjects had been sick a median of 12.5 years, forgetfulness on the one hand or recall bias on the other might affect answers concerning onset or course. However, research on memory shows that events of great importance to a person are much more likely to be remembered accurately (52, 155, 168) than otherwise. For many patients, ME/CFS is a life-changing event so patients often pay extra attention to their condition. In fact, some patients keep extensive notes and even computerized worksheets documenting their symptoms, treatments, and other factors. The agreement between much of what our subjects describe and what other studies found also testifies to memory issues perhaps being less of a concern. Finally, since we attempted to ask about a broad range of subjects, we had to cut down on details to obtain a high survey response rate.

To overcome or reduce these limitations in the future, research should attempt to recruit subjects from various settings (e.g., from the community, primary and specialty clinics), employ the CCC and/or NAM criteria during subject recruitment, gather information prospectively rather than retrospectively, and complement subject-reported accounts with third-party reports (e.g., medical records) and/or objective measures where possible. Areas which would have benefitted from greater detail include which documented infections preceded ME/CFS, what treatments specifically helped with which symptoms, and how ME/CFS symptoms might vary depending on which stage of pregnancy, menstrual cycle, or menopause a woman is occupying.

## Conclusion

This paper gives a broad epidemiologic overview of one ME/CFS cohort in the United States. While our findings concerning onset, course, function, co-morbid conditions, and family history support those of prior studies, by examining these topics together, we were able to interpret our findings within the complicated context of this condition and offer unique insights into how epidemiologic data can be utilized to inform both clinical care and improve future research. We also contribute new information about how ME/CFS symptoms change longitudinally and with events associated with the female reproductive system throughout a woman's life. Finally, we advance hypotheses centered around the human stress response system, autonomic nervous system, and autoimmune mechanisms to explain the similar yet heterogenous elements of ME/CFS.

In the future, we hope to investigate the relationship between clinical characteristics identified in this study and biomarkers, how epidemiological features may vary contingent on different case definitions, and the influence of human leukocyte antigens on ME/CFS initiation and perpetuation. We also hope that other researchers will verify the findings in this paper and probe further into the areas and issues we have identified.

## Data Availability Statement

The datasets for this manuscript are not publicly available because data were collected confidentially, for specific purposes, with the informed consent of the participants. Requests to access the datasets should be directed Alyssa Aguilar at aalyssa@stanford.edu or the Stanford University Institutional Review Board at irbeducation@lists.stanford.edu.

## Ethics Statement

This study was carried out in accordance with the recommendations of United States Federal Policy for the Protection of Human Subjects (known as the Common Rule, 45 CFR part 46) and the Belmont Report with written informed consent from all subjects. All subjects gave written informed consent in accordance with the Declaration of Helsinki. The protocol was approved by the Stanford University Institutional Review Board (Review Panel 3, Protocol 24244).

## Author Contributions

LC, IV, and JM conceived and designed the study, JM obtained funding and supervised the study. JM and IV recruited subjects and collected data. LC and DG analyzed the data and performed the statistical analyses. LC wrote the first draft of the manuscript. All authors contributed to manuscript revision, read, and approved the submitted version.

### Conflict of Interest Statement

The authors declare that the research was conducted in the absence of any commercial or financial relationships that could be construed as a potential conflict of interest.

## Footnotes

^1^https://projectredcap.org/

^2^http://condor.depaul.edu/ljason/cfs/measures.html

^3^http://www.graphpad.com/quickcalcs/
